# Artemisinins inhibit oral candidiasis caused by *Candida albicans* through the repression on its hyphal development

**DOI:** 10.1038/s41368-023-00245-0

**Published:** 2023-09-12

**Authors:** Xiaoyue Liang, Ding Chen, Jiannan Wang, Binyou Liao, Jiawei Shen, Xingchen Ye, Zheng Wang, Chengguang Zhu, Lichen Gou, Xinxuan Zhou, Lei Cheng, Biao Ren, Xuedong Zhou

**Affiliations:** 1 State Key Laboratory of Oral Diseases & National Center for Stomatology & National Clinical Research Center for Oral Diseases & West China Hospital of Stomatology, Sichuan University, Chengdu, China; 2 State Key Laboratory of Oral Diseases & National Center for Stomatology & National Clinical Research Center for Oral Diseases & Department of Operative Dentistry and Endodontics, West China Hospital of Stomatology, Sichuan University, Chengdu, China

**Keywords:** Antifungal agents, Drug development

## Abstract

*Candida albicans* is the most abundant fungal species in oral cavity. As a smart opportunistic pathogen, it increases the virulence by switching its forms from yeasts to hyphae and becomes the major pathogenic agent for oral candidiasis. However, the overuse of current clinical antifungals and lack of new types of drugs highlight the challenges in the antifungal treatments because of the drug resistance and side effects. Anti-virulence strategy is proved as a practical way to develop new types of anti-infective drugs. Here, seven artemisinins, including artemisinin, dihydroartemisinin, artemisinic acid, dihydroartemisinic acid, artesunate, artemether and arteether, were employed to target at the hyphal development, the most important virulence factor of *C. albicans*. Artemisinins failed to affect the growth, but significantly inhibited the hyphal development of *C. albicans*, including the clinical azole resistant isolates, and reduced their damage to oral epithelial cells, while arteether showed the strongest activities. The transcriptome suggested that arteether could affect the energy metabolism of *C. albicans*. Seven artemisinins were then proved to significantly inhibit the productions of ATP and cAMP, while reduced the hyphal inhibition on *RAS1* overexpression strain indicating that artemisinins regulated the Ras1-cAMP-Efg1 pathway to inhibit the hyphal development. Importantly, arteether significantly inhibited the fungal burden and infections with no systemic toxicity in the murine oropharyngeal candidiasis models in vivo caused by both fluconazole sensitive and resistant strains. Our results for the first time indicated that artemisinins can be potential antifungal compounds against *C. albicans* infections by targeting at its hyphal development.

## Introduction

*Candida albicans* is one of the most common fungal species from human mycobiome.^1,2^ It can colonize at multiple body sites, such as oral cavity, skin, gastrointestinal and vaginal tracts.^3,4^ The fungal infectious diseases caused by *C. albicans* have increased fast in recent years and are one of major causes of death in intensive care unit, especially among the immunocompromised populations, including HIV infected patients, cancer patients after the radiation and chemotherapy, organ transplantation patients, etc.^5–7^ *C. albicans* can induce the superficial infection in skin or oral cavity, but it can also cause the systematic infection, such as candidemia.^8^ Oral candidiasis is the most common fungal infectious disease in oral cavity, especially in elderly population, newborn, denture wearers, AIDs patients and head neck cancer patients, while *C. albicans* is the major pathogenic agent.^9,10^ *C. albicans* can damage the oral mucosal epithelial barriers to induce the mucosal infection and may even cause the mucosal malignancy to promote the development of oral cancer.^9,11–13^

During the infectious process, the hyphal development of *C. albicans* is the most important virulent factors.^4,14^ The hyphal forms of *C. albicans* can enhance the adhesion to oral epithelial cells by produce adhesins, such as agglutinin-like sequence (Als) family of proteins.^11,15,16^ It also penetrates the oral epithelial cells through the mechanical damage and the secretion of fungal toxin, such as candidalysin.^17–19^ Moreover, the hyphae promote the resistance of *C. albicans* to host antifungal immunity by inducing the immune escape from the immune cells, such as macrophages and neutrophils.^11,20–22^ Several pathways have been identified to involve in the regulation of *C. albicans* hyphal development,^14^ including Cek MAPK (mitogen-activated protein kinase) pathway,^23^ pH-response pathway,^24^ Hog MAPK pathway,^25,26^ Tup1-mediated pathway^27^ and Ras1-cAMP-Efg1 pathway.^28^ The Ras1-cAMP-Efg1 pathway plays key roles in the morphological development of *C. albicans* for the response to energy metabolism, glycogen biosynthesis, mitochondrial metabolism, etc.^29–32^ Briefly, Ras1 can activate the adenylyl cyclase (Cyr1) to hydrolyze the ATP to produce cAMP.^33^ cAMP then activates the protein kinase A, including Tpk1 and Tpk2 to regulate the activities of the transcription factor Efg1.^34,35^ Efg1 furtherly regulates the expression of Ume6, a master regulator of hypha associated genes.^36–38^ The cAMP level in *C. albicans* cells is the key signal for the activation of Ras1-cAMP-Efg1 pathway.^39,40^ This pathway is also a promising target for the development of new types of antifungal drugs due to its important roles in the regulation of fungal virulence and conservatism in different fungal pathogens.^41–43^

There are three major types of antifungal drugs in clinical, including azoles, polyenes and echinocandins.^44^ Azoles targets at the Erg11 from the ergosterol biosynthesis pathway of *C. albicans* to affect the cell membrane and accumulate toxic sterols.^45^ Polyenes can directly bind to the ergosterol to damage the cell membrane of *C. albicans*.^46^ Echinocandins inhibit the enzyme activity of 1,3-β-D-glucan synthase to block the cell wall biosynthesis.^47,48^ However, due to the lack of new antifungal drugs, the increased resistance has seriously challenged the clinical antifungal treatments.^49–51^ In 2021, FDA has approved a novel class of antifungal drug for vulvovaginal candidiasis named “Ibrexafungerp”, a triterpenoids compound, but it has the similar action with echinocandins targeting at 1,3-β-D-glucan synthase with different binding site.^52,53^ Therefore, new types of antifungal drugs with novel mechanisms are needed emergently.^43,54^ In recent years, anti-virulence therapy has been proved to be a practical and promising strategy for infectious diseases by reducing the inductions of drug resistance, toxicities and effects on the balance of human microbiome.^55,56^ The anti-virulence therapy can block the infectious process of pathogens and their interactions with the host without directly killing or affecting their growth.^55,57^ Traditional Chinese Medicines (TCMs) are efficacious resources for the development of anti-virulence drugs due to its abundant structures, low toxicities, multiple bioactivities.^58,59^

In our previous study, we found that artemisinin, one of the most famous TCMs against malaria, can increase the ergosterol levels of the cell membrane to synergize with polyenes antifungal drug amphotericin B by screening different compounds from TCMs.^60^ Artemisinin enhanced the binding of amphotericin B with *C. albicans* and increased its activities against oral candidiasis.^60^ We also found that artemisinin might affect the hyphal formation of *C. albicans* in that study, however, the actions of other artemisinin analogs, the mechanisms and the therapeutic effectiveness against oral candidiasis of artemisinins alone are not clear. Therefore, with the aims to investigate the actions of artemisinins on the most important virulent factor hyphal formation and the mechanisms against oral candidiasis, seven artemisinins, including artemisinin, dihydroartemisinin, artemisinic acid, dihydroartemisinic acid, artesunate, artemether and arteether, were employed to evaluate their actions and mechanisms on the hyphal development of both azole susceptible and resistant *C. albicans* strains.

## Results

### Artemisinins inhibited the hyphal development of *C. albicans*

**Fig. 1:**
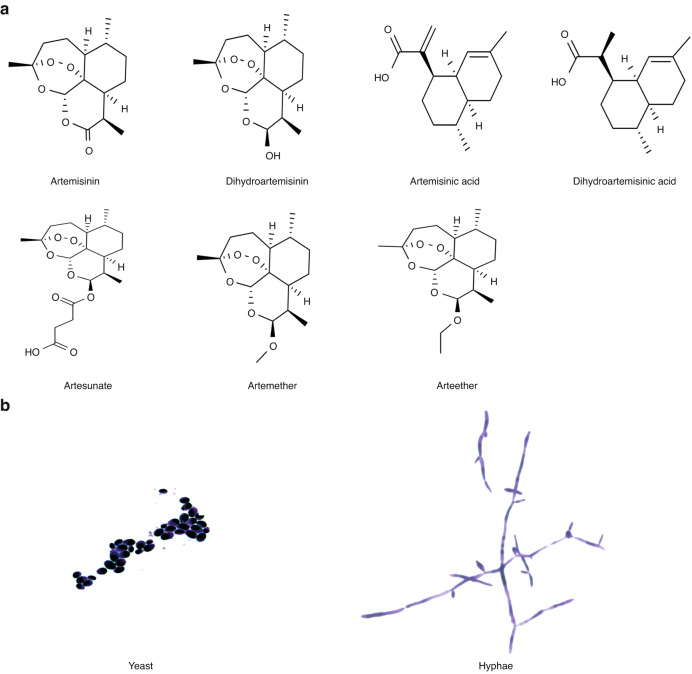
Seven artemisinins and different forms of *C. albicans* **a** Molecular structural formulas of seven artemisinins; **b** Yeast and hphae forms of *C. albicans*

**Fig. 2:**
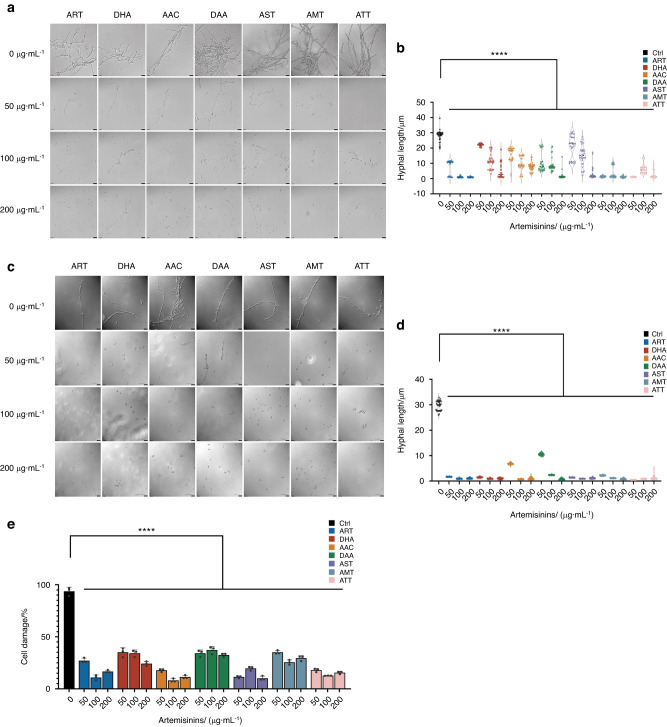
Artemisinins inhibited the hyphal formation of *C. albicans* and its pathogenesis against oral epithelial cells **a** The DIC images of the hyphal formation of strain SC5314 in RPMI 1640 medium treated by 0 (DMSO control), 50, 100 and 200 μg·mL^−1^ artemisinins; **b** The hyphal length of strain SC5314 treated by 0 (DMSO control), 50, 100 and 200 μg·mL^−1^ artemisinins; **c** The DIC images of the hyphal formation of strain SC5314 in spider medium treated by 0 (DMSO control), 50, 100 and 200 μg·mL^−1^ artemisinins; **d** The hyphal length of strain SC5314 treated by 0 (DMSO control), 50, 100 and 200 μg·mL^−1^ artemisinins; **e** The damage rates of oral epithelial cells infected by *C. albicans* with the treatments of 0 (DMSO control), 50, 100 and 200 μg·mL^−1^ artemisinins. ****P < 0.000 1

Seven artemisinins (Fig. 1a), including artemisinin (ART), dihydroartemisinin (DHA), artemisinic acid (AAC), dihydroartemisinic acid (DAA), artesunate (AST), artemether (AMT) and arteether (ATT), were employed to evaluate their effects on the growth and morphological development of *C. albicans* (Fig. 1b). Firstly, we tested their growth inhibitory activities on laboratory standard strain *C. albicans* SC5314 (ATCC MYA − 2876). *C. albicans* SC5314 was sensitive to fluconazole (FLC, MIC = 0.25 μg·mL^−1^), but all of the seven artemisinins showed no growth inhibitory activities (MICs > 200 μg·mL^−1^) (Table 1). Then, the effects on the hyphal development of *C. albicans* were tested in RPMI 1640 and spider media. *C. albicans* SC5314 formed typical hypha in RPMI 1640 and spider media (Fig. 2a, c), while 50, 100 and 200 μg·mL^−1^ of seven artemisinins showed strong inhibitory capabilities on the hyphal formation at a dose dependent manner (Fig. 2b, d), indicating that artemisinins can inhibit the virulence of *C. albicans* without the effects on the growth. To further confirm whether the hyphal inhibition on *C. albicans* SC5314 will repress its infectious capacity, its induction on oral epithelial cell damage was then measured. *C. albicans* SC5314 significantly induced the damage of HOK cells, but all the artemisinins could remarkably reduce the cell damage (Fig. 2e), indicating that artemisinins inhibited the infectious virulence of *C. albicans* by blocking the hyphal formation.

**Table 1:** The inhibitory effects of artemisinins and fluconazole against *C. albicans* SC5314 and 3 clinical isolates

Compounds	MIC/(μg·mL^−1^)			
	SC5314	CCC-39	CCC-32	CCC-80
ART	>200	>200	>200	>200
DHA	>200	>200	>200	>200
AAC	>200	>200	>200	>200
DAA	>200	>200	>200	>200
AST	>200	>200	>200	>200
AMT	>200	>200	>200	>200
ATT	>200	>200	>200	>200
FLC	0.25	0.25	4	>100

### Artemisinins inhibited the hyphal development of clinical azole resistant *C. albicans* isolates

**Fig. 3:**
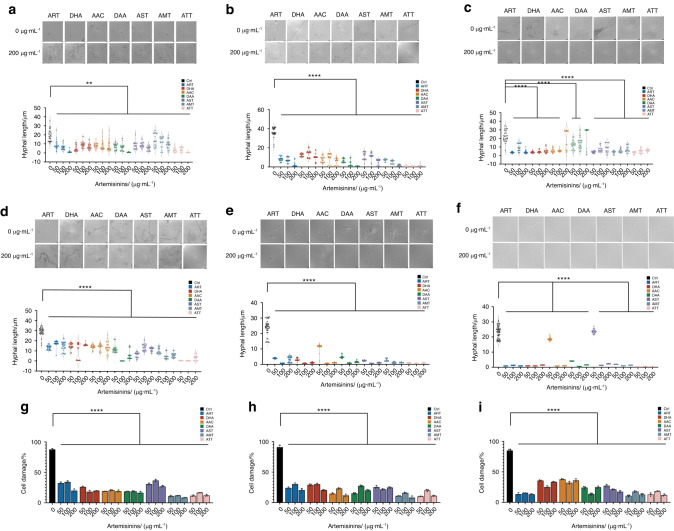
Artemisinins inhibited the hyphal formation of *C. albicans* clinical isolates and their pathogenesis against oral epithelial cells **a** The DIC images of the hyphal formation treated by 0 (DMSO control) and 200 μg·mL^−1^ artemisinins and length of strain CCC-32 in RPMI 1640 medium treated by 0 (DMSO control), 50, 100 and 200 μg·mL^−1^ artemisinins; **b** The DIC images of the hyphal formation treated by 0 (DMSO control) and 200 μg·mL^−1^ artemisinins and length of strain CCC-32 in spider medium treated by 0 (DMSO control), 50, 100 and 200 μg·mL^−1^ artemisinins; **c** The DIC images of the hyphal formation treated by 0 (DMSO control) and 200 μg·mL^−1^ artemisinins and length of strain CCC-39 in RPMI 1640 medium treated by 0 (DMSO control), 50, 100 and 200 μg·mL^−1^ artemisinins; **d** The DIC images of the hyphal formation treated by 0 (DMSO control) and 200 μg·mL^−1^ artemisinins and length of strain CCC-39 in spider medium treated by 0 (DMSO control), 50, 100 and 200 μg·mL^−1^ artemisinins; **e** The DIC images of the hyphal formation treated by 0 (DMSO control) and 200 μg·mL^−1^ artemisinins and length of strain CCC-80 in RPMI 1640 medium treated by 0 (DMSO control), 50, 100 and 200 μg·mL^−1^ artemisinins; **f** The DIC images of the hyphal formation treated by 0 (DMSO control) and 200 μg·mL^−1^ artemisinins and length of strain CCC-80 in spider medium treated by 0 (DMSO control), 50, 100 and 200 μg·mL^−1^ artemisinins; **g** The damage rates of oral epithelial cells infected by *C. albicans* CCC-32 with the treatments of 0 (DMSO control), 50, 100 and 200 μg·mL^−^^1^ artemisinins; **h** The damage rates of oral epithelial cells infected by strain CCC-39 with the treatments of 0 (DMSO control), 50, 100 and 200 μg·mL^−1^ artemisinins; **i** The damage rates of oral epithelial cells infected by strain CCC-80 with the treatments of 0 (DMSO control), 50, 100 and 200 μg·mL^−1^ artemisinins. **P < 0.01; ****P < 0.000 1

Furtherly, clinical *C. albicans* isolates including the azole resistant ones were employed to validate the actions of artemisinins on clinical drug resistant strains. CCC-39 was FLC sensitive isolate (MIC = 0.25 μg·mL^−1^), while CCC-32 (MIC = 4 μg·mL^−1^) and CCC-80 (MICs > 100 μg·mL^−1^) were FLC resistant isolates (Table 1). Seven artemisinins also showed no growth inhibitory effects on these clinical strains (MICs > 200 μg·mL^−1^) (Table 1). However, these artemisinins at 50, 100 and 200 μg·mL^−1^ also significantly inhibited the hyphal formation of all the clinical isolates at a dose dependent manner in both RPMI 1640 (Fig. 3 and S1A, C, E) and spider media (Fig. 3 and S1B, D, F). The infection on oral epithelial cells were then measured. All artemisinins were able to inhibit the oral epithelial cell damage caused by clinical isolates including the azole resistant ones (Fig. 3g–i). Interestingly, it was seemed that artemisinins showed stronger inhibitory activities against the hyphal development and infections to oral epithelial cells of FLC resistant isolates at some dosages (Fig. 3 and S1), indicating that artemisinins inhibit the virulence of both azole sensitive and resistant strains, which highlighted their potential applications against the infections caused by resistant *C. albicans*. Among the seven artemisinins, ATT was one of the most active compounds against the hyphal formation of the clinical isolates (Fig. 3 and S1) and it was then chosen for the further mechanism investigation and in vivo validation.

### Arteether affected the energy metabolism pathway of *C. albicans*

**Fig. 4:**
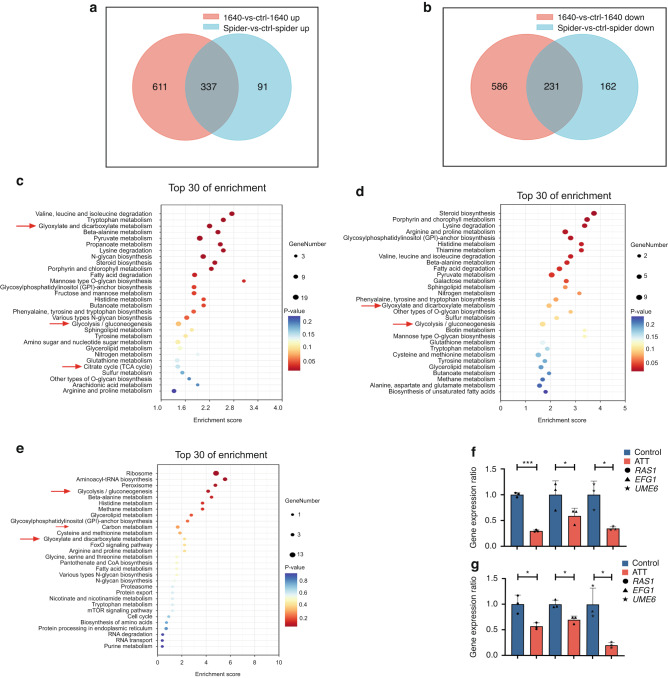
Arteether (ATT) affected the energy metabolism of *C. albicans* **a** The Venn diagram for the numbers of upregulated genes of *C. albicans* SC5314 in both RPMI 1640 and spider media treated with 200 μg·mL^−1^ ATT compared to the DMSO treated control group; **b** The Venn diagram for the numbers of downregulated genes of *C. albicans* SC5314 in both RPMI 1640 and spider media treated with 200 μg·mL^−1^ ATT compared to the DMSO treated control group; **c** KEGG enrichment analysis of differentially expressed genes of SC5314 in RPMI 1640 medium with 200 μg·mL^−1^ ATT treatment compared to the DMSO treated control group (Top 30) and the carbohydrate metabolism related pathways were indicated by red arrows; **d** KEGG enrichment analysis of differentially expressed genes of SC5314 in spider medium treated with 200 μg·mL^−1^ ATT compared to the DMSO treated control group (Top 30) and the carbohydrate metabolism related pathways were indicated by red arrows; **e** KEGG enrichment analysis of the collective differentially expressed genes of SC5314 in both RPMI 1640 and spider media with 200 μg·mL^−1^ ATT treatment compared to the DMSO treated control group (Top 30) and the carbohydrate metabolism related pathways were indicated by red arrows; **f** The different expressions of the key genes from the Ras1-cAMP-Efg1 pathway in RPMI 1640 medium according to the RNA sequencing data; **g** The different expressions of the key genes from the Ras1-cAMP-Efg1 pathway in spider medium according to the RNA sequencing data. *P < 0.05; ***P < 0.001

To furtherly investigate that how the artemisinins inhibited the hyphal formation of *C. albicans*, the transcriptomes of *C. albicans* in RPMI 1640 and spider media with or without the treatment of 200 μg·mL^−1^ ATT were then sequenced, respectively. ATT significantly affected the transcriptome of *C. albicans* in both RPMI 1640 and spider media (Fig. S2A and B). 337 genes were significantly upregulated (Fig. 4a), while 231 genes were significantly downregulated (Fig. 4b) simultaneously in these two media. In line with the inhibition of artemisinins on the hyphal formation of *C. albicans*, several genes related the hyphal development were changed after the treatment of ATT (Fig. S2C and D). To identity the hyphal regulation key pathways which were affected by ATT, the KEGG enrichment analysis was performed. ATT significantly affected the metabolism of *C. albicans*, especially the carbon source metabolism in RPMI 1640 medium (Fig. 4c), spider medium (Fig. 4d) and their collectively enriched pathways (Fig. 4e), suggesting that ATT might regulate the energy metabolism to inhibit the hyphal formation of *C. albicans*. As the hyphal regulation pathway Ras1-cAMP-Efg1 is the main to response the energy metabolism, the genes from this pathway were then analyzed. The key genes from this pathway including *RAS1*, *EFG1* and *UME6* were significantly reduced by ATT in both RPMI 1640 (Fig. 4f) and spider (Fig. 4g) media, indicating that ATT might inhibit the Ras1-cAMP-Efg1 pathway to repress the hyphal formation of *C. albicans*.

### Artemisinins reduced the ATP and cAMP levels to inhibit the Ras1-cAMP-Efg1 pathway of *C. albicans*

**Fig. 5:**
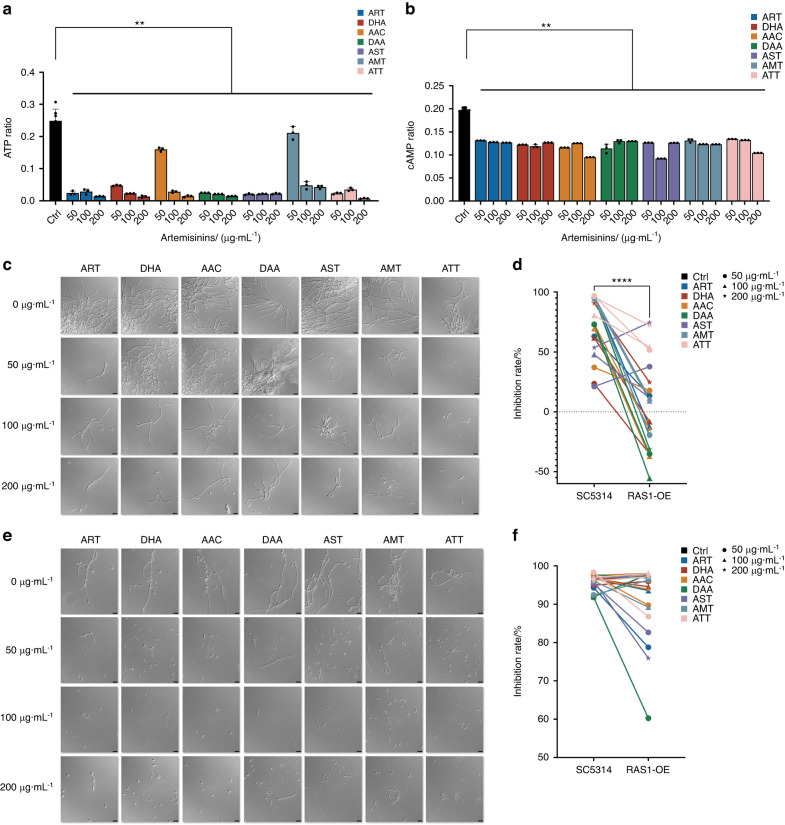
Artemisinins inhibited the Ras1-cAMP-Efg1 hyphal regulation pathway **a** Artemisinins inhibited the ATP production of *C. albicans* SC5314 at 0 (DMSO control), 50, 100 and 200 μg·mL^−1^ **b** Artemisinins inhibited the ATP production of *C. albicans* SC5314 at 0 (DMSO control), 50, 100 and 200 μg·mL^−1^; **c** The DIC images of the hyphal formation of *RAS1* overexpression strain RAS1-OE in RPMI 1640 medium treated by 0 (DMSO control), 50, 100 and 200 μg·mL^−1^ artemisinins; **d** The comparison of the hyphal inhibition rate between strains SC5314 and RAS1-OE in RPMI 1640 medium after the treatments of 50, 100 and 200 μg·mL^−1^ artemisinins; **e** The DIC images of the hyphal formation of *RAS1* overexpression strain RAS1-OE in spider medium treated by 0 (DMSO control), 50, 100 and 200 μg·mL^−1^ artemisinins; **f** The comparison of the hyphal inhibition rate between strains SC5314 and RAS1-OE in spider medium after the treatments of 50, 100 and 200 μg·mL^−1^ artemisinins. **P < 0.01; ****P < 0.000 1

To furtherly confirm the inhibitory actions of artemisinis on Ras1-cAMP-Efg1 pathway of *C. albicans*, the levels of main products ATP and cAMP from this pathway were then measured. Both ATT and other analogs significantly reduced the levels of ATP (Fig. 5a) and cAMP (Fig. 5b). The *RAS1* overexpression strain RAS1-OE were then employed to further validate the activities of artemisinins on this pathway. The *RAS1* overexpression significantly reduced the inhibitory effects of seven artemisinins in both RPMI 1640 (Fig. 5c, d) and spider (Fig. 5e, f) media compared with *C. albicans* SC5314, the wild type strain, especially under the treatment of low dosages of artemisinisns. The inhibitory actions of artemisinins on ATP and cAMP production, and reduced activities on RAS1-OE strain proved that artemisinins could inhibit the Ras1-cAMP-Efg1 pathway to repress the hyphal formation of *C. albicans* and block its pathogenic virulence (Fig. 5).

### Arteether treated the mouse oropharyngeal candidiasis caused by azole sensitive and resistant *C. albicans*

**Fig. 6:**
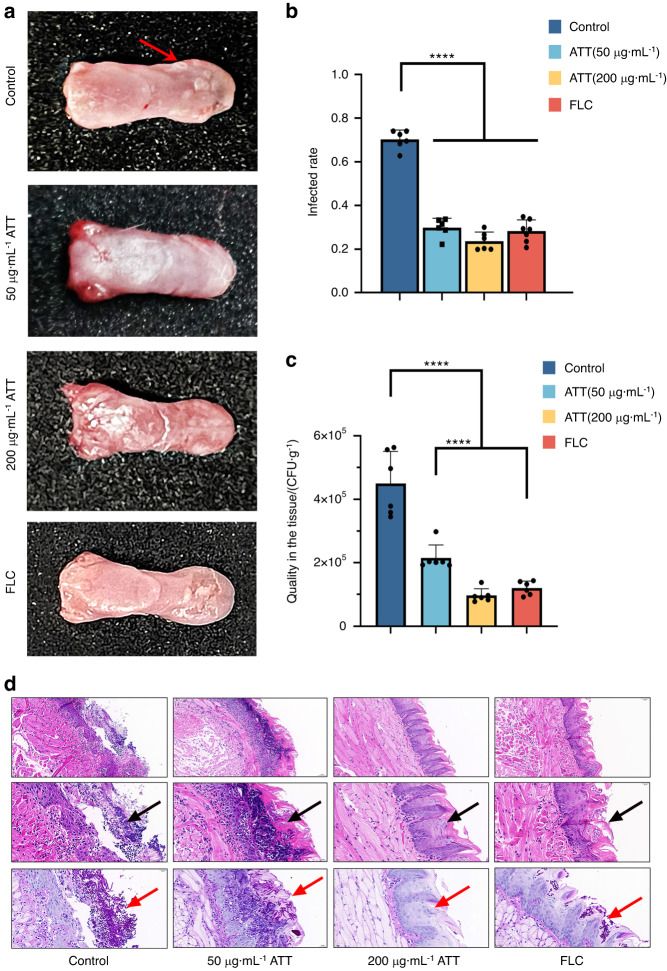
Arteether (ATT) inhibited the oral candidiasis caused by *C. albicans* SC5314 **a** The images and white patches (red arrow) of the mouse tongues infected by SC5314 after the treatments of DMSO control, 50 and 200 μg·mL^−1^ ATT, and 0.25 μg·mL^−1^ FLC; **b** Qualities of the white patches infectious areas caused by SC5314 treated by DMSO control, 50 and 200 μg·mL^−1^ ATT, and 0.25 μg·mL^−1^ FLC; **c** Qualities of the *C. albicans* colonization in the infected tongues with the treatments of DMSO control, 50 and 200 μg·mL^−1^ ATT, and 0.25 μg·mL^−1^ FLC; **d** The HE (up row: 100× magnification; middle row: 400× magnification) and PAS staining of epithelium of the infected tongues after treated by DMSO control, 50 and 200 μg·mL^−1^ ATT, and 0.25 μg·mL^−1^ FLC. The infiltrating inflammatory cells were indicated by the black arrow and the invading hyphae were indicated by the red arrow. *n* = 6, per group; ****P < 0.000 1

The mouse oropharyngeal candidiasis models caused by both azole sensitive and resistant strains were then employed to evaluate the activity ATT against oral candidiasis in vivo, while fluconazole was served as clinical drug control. In the oropharyngeal candidiasis caused by azole sensitive strain *C. albicans* SC5314 (*n* = 6, per group), both FLC and ATT significantly reduced the infections on the tongues (Fig. 6a). The treatments of ATT at 50 and 200 μg·mL^−1^ significantly repressed the white patches infectious areas compared to the untreated groups and were similar with that from the clinical antifungal drug FLC treated group (Fig. 6b). ATT also significantly reduced the fungal burden at a dose dependent manner and was similar with the FLC treated group at 200 μg·mL^−1^ (Fig. 6c). According to the HE staining results in Fig. 6d, ATT also reduced the infiltrating inflammatory cells in the tongues. In terms of the PAS staining results, ATT significantly inhibited the hyphal forms of *C. albicans* in the tongues at 50 μg·mL^−1^ (Fig. 6d). Seldom *C. albicans* cells were observed in the ATT 200 μg·mL^−1^ treated group, while some yeast forms of *C. albicans* were still observed in the FLC treated group (Fig. 6d), indicating that ATT could inhibit the hyphal formation of *C. albicans* in vivo to reduce its host colonization and the infectious pathogenesis of *C. albicans*.

**Fig. 7:**
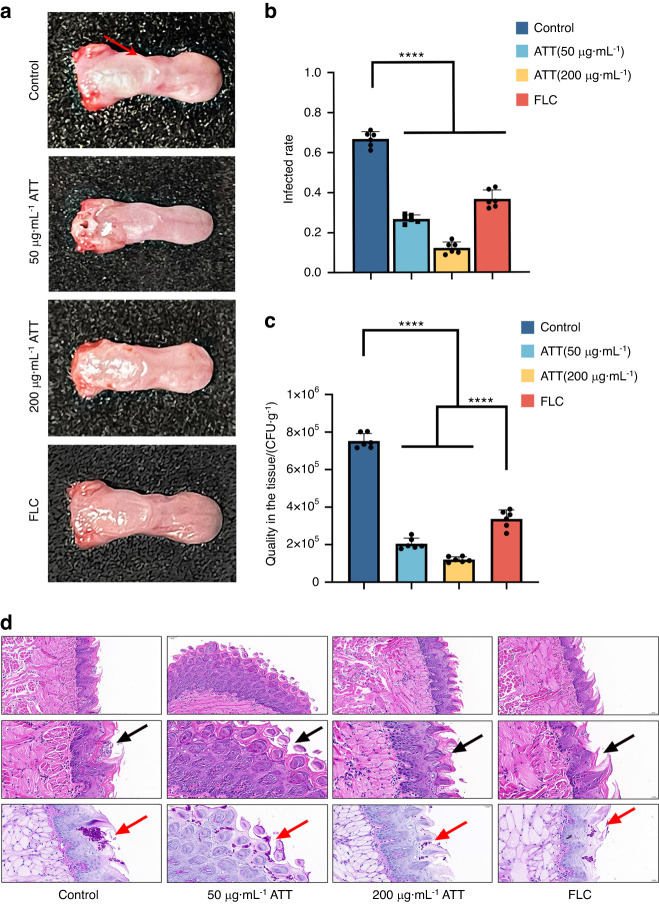
Arteether (ATT) inhibited the oral candidiasis caused by clinical resistant isolate CCC-80 **a** The images and white patches (red arrow) of the mouse tongues infected by CCC-80 after the treatments of DMSO control, 50 and 200 μg·mL^−1^ ATT, and 0.25 μg·mL^−1^ FLC; **b** Qualities of the white patches infectious areas caused by CCC-80 treated by DMSO control, 50 and 200 μg·mL^−1^ ATT, and 0.25 μg·mL^−1^ FLC; **c** Qualities of the *C. albicans* colonization in the infected tongues with the treatments of DMSO control, 50 and 200 μg·mL^−1^ ATT, and 0.25 μg·mL^−1^ FLC; **d** The HE (up row: 100× magnification; middle row: 400× magnification) and PAS staining of epithelium of the infected tongues after treated by DMSO control, 50 and 200 μg·mL^−1^ ATT, and 0.25 μg·mL^−1^ FLC. The infiltrating inflammatory cells were indicated by the black arrow and the invading hyphae were indicated by the red arrow. *n* = 6, per group; ***P < 0.001; ****P < 0.000 1

To investigate the activities of ATT on the fungal infections caused by drug resistant strain, the mouse oropharyngeal candidiasis model caused by CCC-80 was constructed (*n* = 6, per group). FLC reduced the therapeutic effects CCC-80 infected tongues compared with that infected by SC5314 based on the white patches infectious areas and fungal burden (Figs. 6, 7a, b and c), however, ATT showed the similar inhibitory activities in CCC-80 induced oropharyngeal candidiasis compared with that infected by SC5314 (Figs. 6, 7a, b and c). ATT also significantly reduced the infiltrating inflammatory cells and *C. albicans* colonization in the tongues at dose dependent manner (Fig. 7d), indicating that ATT was also capable to treat the oral candidiasis caused by azole resistant strain through the inhibition on the hyphal formation to reduce the infectious virulence.

**Fig. 8:**
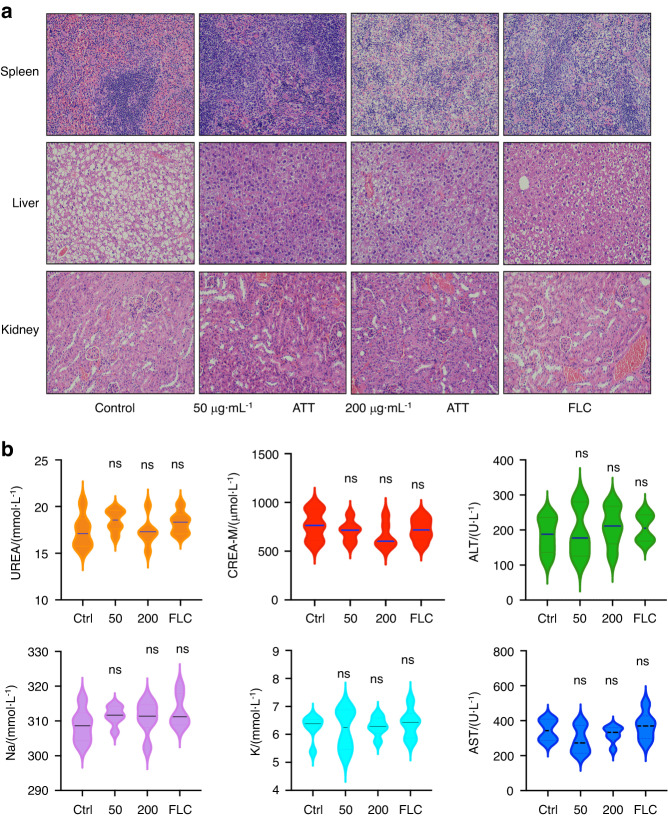
No significant systemic toxicity of ATT treatments applied locally in oral cavity **a** The HE staining of the major organs including liver, kidney and spleen after the local administration of DMSO control, 50 and 200 μg·mL^−1^ ATT, and 0.25 μg·mL^−1^ FLC in mouse oral cavity; **b** The concentration of blood biochemical indicators, including blood urea (UREA), alanine aminotransferase (ALT), aspartate aminotransferase (AST), and creatinine (CREA), Na^+^ and K^+^, after the local administration of DMSO control, 50 and 200 μg·mL^−1^ ATT, and 0.25 μg·mL^−1^ FLC in mouse oral cavity. *n* = 6, per group; ns no significance

To evaluate the systematic toxicities of ATT, the HE staining of the major organs (*n* = 6, per group), including spleen, liver and kidney and the blood biochemical indicators, including blood urea (UREA), alanine aminotransferase (ALT), aspartate aminotransferase (AST), and creatinine (CREA), Na^+^ and K^+^, were measured. The HE staining results from the major metabolic organs showed that the application of ATT led to no obvious systematic toxicity on major organs (Fig. 8a), while the levels of the blood biochemical indicators also represented no significant changes from the ATT treated groups (Fig. 8b), indicating that ATT could show strong therapeutic effects on oral candidiasis without significant systematic toxicities.

## Discussion

The increased fungal infections, especially in immunocompromised populations, have serious impacts on human health and even lives.^61^ However, there are no antifungal drugs with new actions for decades in clinical and only in 2021, Ibrexafungerp, a kind of triterpenoids, has been approved for vulvovaginal candidiasis with the similar action as echinocandins on cell wall biosynthesis at a different binding site.^53^ The lack of new type of antifungal drugs with novel mechanisms and overuse of current clinical antifungal drugs have challenged current treatments and increased the fungal infections caused by drug resistant strains, and even new natural resistant species have been identified in clinical, such as *C. auris*^62,63^ and *C. vulturna*.^64^ Therefore, it’s urgent to find new ways to develop novel antifungal drugs.

The research of anti-virulence strategy has increased rapidly in recent years and this strategy has been proved its advantages in the reductions of drug resistance, toxicities and effects on human microbiome, and in the therapeutic effectiveness on the infections caused by resistant strains. Unlike the traditional anti-infective drug to directly kill the pathogens or inhibit their growth, the anti-virulence compounds are designed to inhibit the pathogenic processes or block their interactions with the host. Many TCMs have showed potential anti-virulence activities.^65–68^

Artemisinins, traditional antimalarial drugs, have also showed different actions on microbial pathogens.^69,70^ Previously, we have revealed for the first time that ART could upregulate the ergosterol biosynthesis pathway to enhance the ergosterol levels and then increase the binding between *C. albicans* cells and polyene antifungals to synergize with amphotericin B against oral candidiasis.^60^ However, how the artemisinins alone act on *C. albicans* is still unknow. By screening seven artemisinins, we firstly found that seven artemisnins could inhibit the hyphal development of both drug sensitive and resistant *C. albicans*, the most important virulence factor, indicating that artemisinins can be potential candidates for anti-fungal virulence drugs.

Several pathways have been identified to regulate the development of *C. albicans* hypha, while the Ras1-cAMP-Efg1 is the key pathway to regulate the hyphal formation to respond the shift of the energy metabolism of *C. albicans* under different stresses.^30,32,39,40^ ATP and cAMP are critical signaling molecules to activate this pathway.^29,32,40^ In this study, we for the first time found the mechanism of artemisinins on *C. albicans* hypha that they could affect the energy metabolism of *C. albicans* to reduce the ATP and cAMP levels and repress the Ras1-cAMP-Efg1 pathway to inhibit the hyphal formation and the pathogenic virulence to both oral epithelial cells and mouse oropharyngeal candidiasis, indicating the new mechanisms of artemisinins on *C. albicans*. Importantly, artemisinins even showed stronger activities on the hyphal development, virulence, and infections of fluconazole resistant strains, indicating that the potential practical application of artemisinins on the *C. albicans* infections even caused by drug resistant strains. Additionally, all of the seven artemisinins could affect the ATP and cAMP levels to inhibit the hyphal formation suggesting that the common structure in these compounds is key to regulate the energy metabolism, while AAC and DAA also showed strong activities on the inhibitions of ATP and cAMP indicating that the endo-peroxide bridge, which is the major molecular portion against malarial parasite,^71,72^ was not the key structure for the actions of artemisinins on *C. albicans*, suggesting new promising bioactivities of artemisinins, especially on microbial pathogens. More analogs could be employed in the further investigations to identify the “core structures” of artemisinins that acting on microbes.

Combined with the synergistic antifungal action of ART with amphotericin B from our previous results^60^ and the anti-fungal virulence activities of artemisinins in this study, artemisinins could be served as new antifungal drug candidates for oral candidiasis even caused by drug resistant *C. albicans*. However, we haven’t found the direct binding targets of artemisinins in *C. albicans* yet, which are highly associated with the ergosterol biosynthesis and Ras1-cAMP-Efg1 hyphal regulation pathway, such as how the artemisinins upregulate the ergosterol pathway but downregulate the energy pathways, how the artemisinins reduce the ATP and cAMP levels and so on. Our next work in the future will focus on the direct binding targets of artemisinins from *C. albcians* and also evaluate their actions on different *C. albicans* infectious models, such as systematic infections and other *Candida* species including *C. auris* etc., to promote the development of artemisnins as new type of antifungal drugs.

In conclusion, for the first time, we found that artemisinins could inhibit the Ras1-cAMP-Efg1 pathway to repress the hyphal formation of *C. albicans*. The hyphal inhibition effects of artemisninins furtherly reduced the infections caused by both drug sensitive and resistant *C. albicans* strains on oral epithelial cells in vitro and oral mucosa in vivo. Our results have highlighted that antifungal virulence is a practical strategy to identify new types of antifungal candidate drugs against clinical drug resistant strains and TCMs, such as artemisinins in this study, were great resource for developing new anti-infection drugs.

## Materials and methods

### Ethics statement

All mouse experiments described in this study were conducted in strict accordance with the guidelines of Ethics Committee of West China Hospital of Sichuan University and the protocols were full approved by this Agency (WCHSIRB-D-2022-568). All efforts were made to minimize suffering and ensure the highest ethical and humane standards.

### Chemicals

Artemisinin (CAS 63968-64-9, Purity 98.00%), Dihydroartemisinin (CAS 71939-50-9, Purity 98.00%), Artemisinic Acid (CAS 80286-58-4, Purity ≥98.00%), DihydroarteMisinic acid (CAS 85031-59-0, Purity ≥98.00%), Artesunate (CAS 88495-63-0, Purity 98.00%), Artemether (CAS 71963-77-4, Purity 98.00%) and Arteether (CAS 75887-54-6, Purity ≥98.00%) were from China Sourcing Biology Technology Co., Ltd. Fluconazole (CAS 86386-73-4, Purity ≥98.00%) was from China BaiLingWei Company. All compounds were dissolved in dimethyl sulfoxide (DMSO, Merck, China) and stored at −20 °C until use.

### Strains and culture conditions

The laboratory standard strain *C. albicans* SC5314 (ATCC MYA-2876) and 3 clinical isolates (CCC-32, CCC-39 and CCC-80) were stored in the State Key Laboratory of Oral Diseases, West China School of Stomatology. *RAS1* overexpression mutant was constructed as described previously.^73^ All strains were maintained on YPD agar medium (1% yeast extract, 2% peptone, 2% glucose and 2% agar) and were stored at -80 °C until use. Single colonies were picked and incubated overnight in YPD broth medium (1% yeast extract, 2% peptone and 2% glucose) at 37 °C.

### Antifungal susceptibility testing

The antifungal activity of drugs was tested according to the Clinical and Laboratory Standards Institute document M27A as previously described.^60,74^ Briefly, overnight cultured *C. albicans* were centrifuged and resuspended in RPMI 1640 medium to achieve a final concentration of 1 × 10^4^ CFU·mL^−1^. The Minimum Inhibitory Concentration (MIC) of the seven compounds was determined using a two-fold serial dilution in 96-well plates. The final concentrations of the seven compounds were set as follows: 200, 100, 50, 25, 12.5, 6.25, 3.125 and 1.5625 µg·mL^−^^1^. DMSO was used as negative control, while fluconazole was used as positive control. The plates were then incubated at 37 °C for 24 h. After incubation, the turbidity of each well was measured by using a Multi-Mode Microplate Reader (SpectraMax iD5, Molecular Devices, LLC.) at an optical density (OD) of 600 nm. The MIC was defined as the lowest concentration at which no fungal growth was observed. All of the experiments were performed in triple.

### *C. albicans* hyphal formation measurement

*C. albicans* hyphal formation was induced in RPMI 1640 and Spider media (Spider medium: 1% Nutrient broth, 1% D-mannitol, 0.2% K_2_HPO_4_). Single colonies of *C. albicans* were picked and suspended in RPMI 1640 and spider media to prepare a fungal suspension. After counting using a hemocytometer, the fungal concentration was adjusted to 1 × 10^5^ CFU·mL^–1^. The prepared fungal suspensions were then added to a 96-well plate. DMSO was used as the negative control, and the seven compounds were added to the wells at concentrations of 200, 100, and 50 μg·mL^−1^, respectively. The 96-well plate was incubated at 37 °C with 5% CO_2_ for 24 h. 10 μL of the culture was taken and placed on a microscope slide. After sealing the slide, hyphal formation was observed under a phase-contrast microscope (DMi8, Leica), while the hyphal length from over 30 cells were calculated according to the ImageJ software (Version 1.8.0.112). The comparison of hyphae inhibitory rates treated by different concentrations of artemisinins on *C. albicans* SC5314 and RAS1-OE were according to the hyphal length medians of the treated groups compared to that of DMSO treated groups.

### Oral epithelial cell damage assay

The oral epithelial cell damage assay was performed by the Roche cytotoxicity detection kit^plus^ as previously described.^75,76^ Briefly, the oral epithelial cell Human Oral Keratinocytes (HOK) isolated from the normal oral mucosa was cultured in a 5% CO_2_ incubator at 37 °C. After 24 h, the cells were digested with trypsin, and passaged. The HOK cells were then transferred into a 96 well plate and cultured overnight. The culture supernatant was then replaced by fresh DMEM medium containing different concentrations of artemisinins at 200 μg·mL^−1^, 100 μg·mL^−1^, 50 μg·mL^−1^ and DMSO, respectively. After incubating at 37 °C and 5% CO_2_ for 24 h, 20% lysis buffer was added to the control wells, while 50% working solution was added to each well, and shake at room temperature in dark for 30 min, then 25% top solution was added to each well according to the manufacturer’s instructions of the kit. After that, the culture supernatants were collected and the absorbance was detected by using a Multi-Mode Microplate Reader (SpectraMax iD5, Molecular Devices, LLC.) at 459 nm.

### Transcriptome sequencing and analysis

The single colony of *C. albicans* SC5314 was picked and cultured in YPD liquid medium at 37 °C overnight. The fungal concentration was adjusted to 1 × 10^8^ CFU·mL^−1^ using RPMI 1640 and spider media, respectively. 200 μg·mL^−1^ of artemether was employed to treat the fungal cells while DMSO was served as control. After 6 h, the fungal cells were collected by high-speed centrifugation, followed by rapid freezing in liquid nitrogen and storage at −80 °C. All of the samples were prepared in triple.

The *C. albicans* transcriptome sequencing and analysis were performed by Shanghai OE Biotech Co., Ltd. Briefly, the Illumina TruSeqTM RNA sample prep Kit method was used for library preparation, while the Illumina NovaSeq 6000 sequencing platform was used to sequence all the mRNA. Sequencing data was subjected to quality control through data statistics and raw data analysis. Using the fastp software, raw reads in fastq format were processed to obtain clean reads for subsequent data analysis. HISAT2 software was employed for reference genome alignment, and gene expression levels (FPKM) were calculated. DESeq2 software was applied for differential gene expression analysis, and differentially expressed genes were subjected to KEGG Pathway enrichment analysis based on the hypergeometric distribution algorithm to identify significantly enriched functional terms. GSEA software was used for gene set enrichment analysis. The sequence data was available online (https://www.ncbi.nlm.nih.gov/sra/PRJNA999911).

### ATP and cAMP extraction and measurement

The extractions and measurements of ATP and cAMP were performed as previously described.^73^ Different concentrations of artemisinins at 50, 100, and 200 μg·mL^−1^ were added to treat the fungal cells for 6 h, while DMSO was served as control. The luminescence and absorbance at 450 nm were measured by using a Multi-Mode Microplate Reader (SpectraMax iD5, Molecular Devices, LLC.). All the experiments were performed in triple.

### Murine oropharyngeal candidiasis model

The murine oropharyngeal candidiasis model was constructed and performed as previously described.^18,73,75,77^ The female BALB/c mice, aged 4–6 weeks and weighing approximately 20 g, provided by the Experimental Animal Center of Sichuan University, were infected by both *C. albicans* SC5314 and CCC-80. The mice were randomly divided into four groups (*n* = 6, per group), including DMSO treated group, 50 μg·mL^−1^ arteether treated group, 200 μg·mL^−1^ arteether treated group and 0.25 μg·mL^−1^ fluconazole treated group, with six mice in each group. Arteether and fluconazole were administered through drinking water. The area of tongue infection was observed and recorded in each group. The tongue tissue was divided into two parts along the longitudinal axis, one part was used for histopathological examination, and the other part was used to measure the fungal burden of *C. albicans*. The organs, including kidney, liver and spleen, and the blood from the mice were also collected for histological analysis and blood biochemistry analysis, including blood urea (UREA), alanine aminotransferase (ALT), aspartate aminotransferase (AST), and creatinine (CREA), Na+ and K+, in Chengdu Aochuang Biotechnology Co., Ltd., respectively.

### Statistical analysis

The statistics of the length of hyphae, damage rates of oral epithelial cells, infectious areas and CFU of mouse models from artemisininis treated groups compared to that from the control groups were analyzed with Ordinary one-way ANOVA and Bonferroni’s multiple comparisons test, while the expressions of the key genes from Ras1-cAMP-Efg1 pathway from arteether treated groups compared to that from the control groups were analyzed with *t* test. The comparison of the hyphae inhibitory rates on SC5314 and RAS1-OE was analyzed with Wilcoxon matched-pairs signed rank test. All the statistical analysis and construction of figures were performed by Graphpad prism (version 9.5.0).

### Supplementary information

Legends for supplementary figures

Supplementary Figure S1

Supplementary Figure S2
