# Supplementary material for: Artemisinins inhibit oral candidiasis caused by Candida albicans through the repression on its hyphal development
Source: Int J Oral Sci. 2023 Sep 12;15:40. doi: 10.1038/s41368-023-00245-0 (PMC10497628; doi:10.1038/s41368-023-00245-0)
Supplement: Supplementary file 1 — Legends for supplementary figures [file 41368_2023_245_MOESM1_ESM.docx]

**Fig. S1. Artemisinins inhibited the hyphal formation of *C. albicans* clinical isolates. A.** The DIC images of the hyphal formation of strain CCC-32 in RPMI 1640 medium treated by 50 and 100 μg/mL artemisinins; **B.** The DIC images of the hyphal formation strain CCC-32 in spider medium treated by 50 and 100 μg/mL artemisinins; **C.** The DIC images of the hyphal formation of strain CCC-39 in RPMI 1640 medium treated by 50 and 100 μg/mL artemisinins; **D.** The DIC images of the hyphal formation of strain CCC-39 in spider medium treated by 50 and 100 μg/mL artemisinins; **E.** The DIC images of the hyphal formation of strain CCC-80 in RPMI 1640 medium treated by 50 and 100 μg/mL artemisinins; **F.** The DIC images of the hyphal formation of strain CCC-80 in spider medium treated by 50 and 100 μg/mL artemisinins.

**Fig. S2. The differentially expressed genes after the treatment of arteether.** **A.** Volcano plot of differentially expressed genes of *C. albicans* SC5314 treated by 200 μg/mL arteether compared to that of the DMSO treated control group in RPMI 1640 medium; **B.** Volcano plot of differentially expressed genes of *C. albicans* SC5314 treated by 200 μg/mL arteether compared to that of the DMSO treated control group in spider medium. **C.** Heatmap of the hyphal development related genes treated by 200 μg/mL arteether compared to that of the DMSO treated control group in spider medium; **D.** Heatmap of the hyphal development related genes treated by 200 μg/mL arteether compared to that of the DMSO treated control group in spider medium.
