# Supplementary figures and images for: Artemisinins inhibit oral candidiasis caused by Candida albicans through the repression on its hyphal development
Source: Int J Oral Sci. 2023 Sep 12;15:40. doi: 10.1038/s41368-023-00245-0 (PMC10497628; doi:10.1038/s41368-023-00245-0)

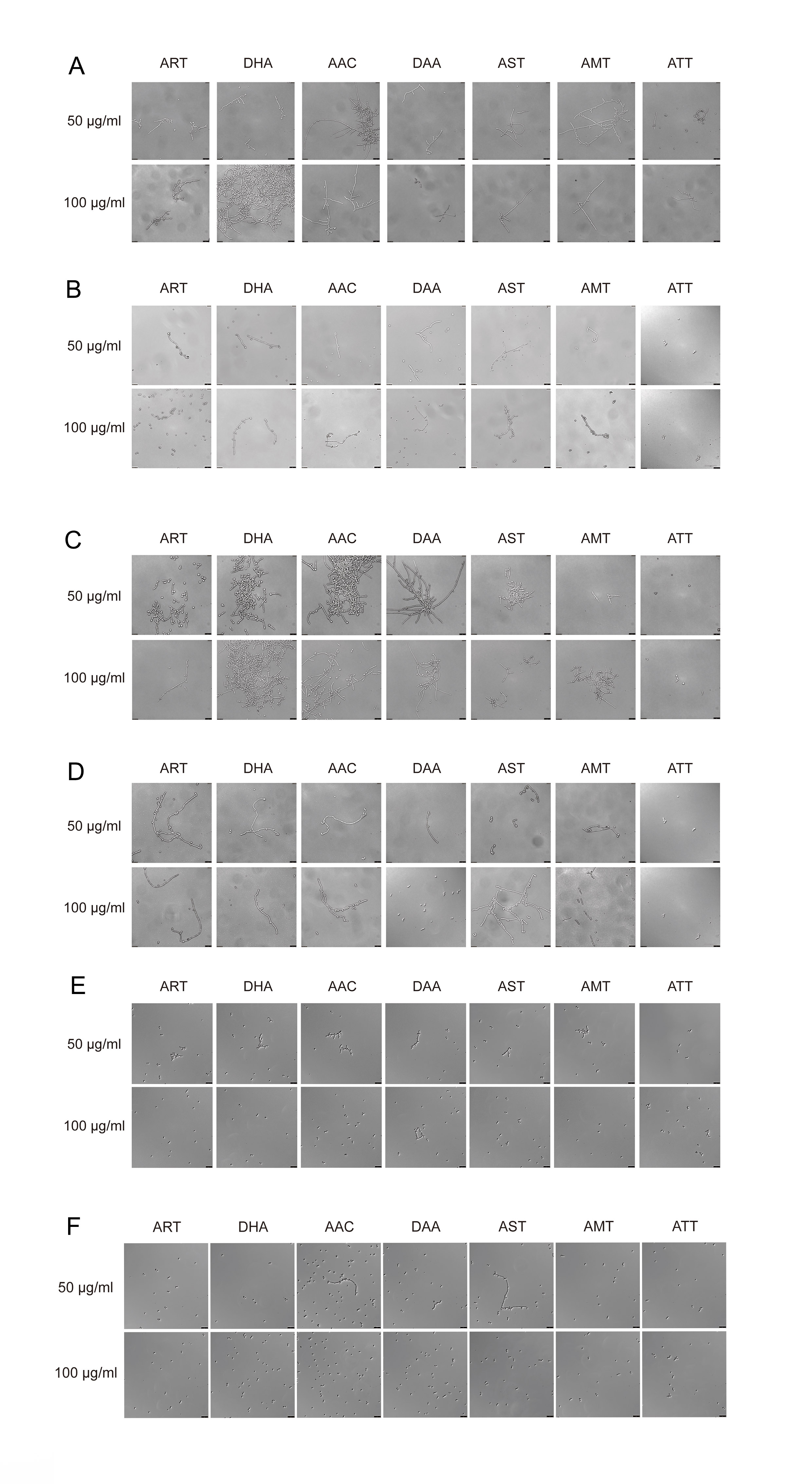

Supplement: Supplementary file 2 — Supplementary Figure S1 [file 41368_2023_245_MOESM2_ESM.jpg]

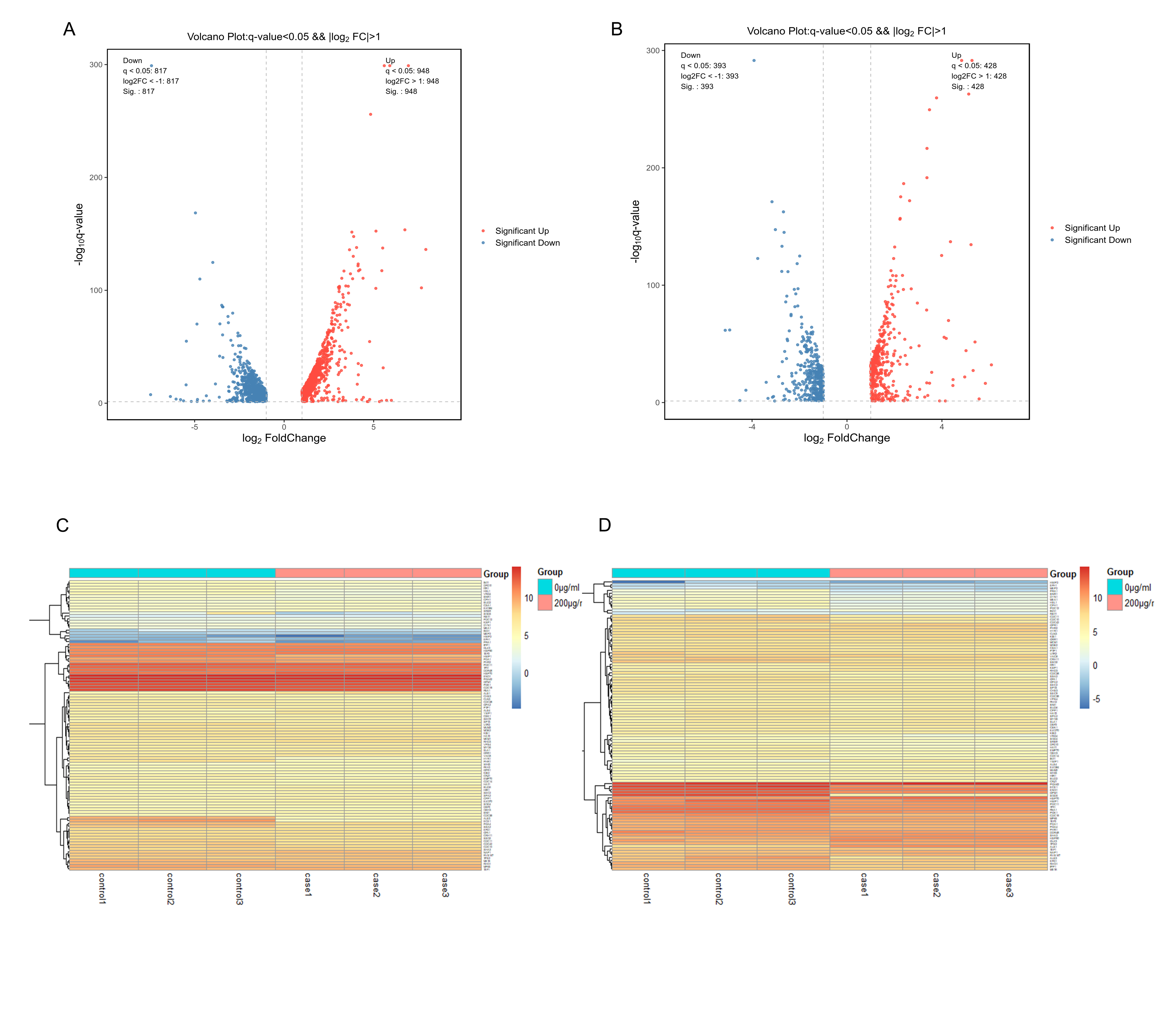

Supplement: Supplementary file 3 — Supplementary Figure S2 [file 41368_2023_245_MOESM3_ESM.jpg]
